# Is There a Regulatory Role of Immunoglobulins on Tissue Forming Cells Relevant in Chronic Inflammatory Lung Diseases?

**DOI:** 10.1155/2011/721517

**Published:** 2011-11-02

**Authors:** Michael Roth

**Affiliations:** Pulmonary Cell Research, Department of Research and Pneumology, University Hospital Basel, 4031 Basel, Switzerland

## Abstract

Epithelial cells, fibroblasts and smooth muscle cells together form and give structure to the airway wall. These three tissue forming cell types are structure giving elements and participate in the immune response to inhaled particles including allergens and dust. All three cell types actively contribute to the pathogenesis of chronic inflammatory lung diseases such as asthma and chronic obstructive pulmonary disease (COPD). Tissue forming cells respond directly to allergens through activated immunoglobulins which then bind to their corresponding cell surface receptors. It was only recently reported that allergens and particles traffic through epithelial cells without modification and bind to the immunoglobulin receptors on the surface of sub-epithelial mesenchymal cells. In consequence, these cells secrete pro-inflammatory cytokines, thereby extending the local inflammation. Furthermore, activation of the immunoglobulin receptors can induce proliferation and tissue remodeling of the tissue forming cells. New studies using anti-IgE antibody therapy indicate that the inhibition of immunoglobulins reduces the response of tissue forming cells. The unmeasured questions are: (i) why do tissue forming cells express immunoglobulin receptors and (ii) do tissue forming cells process immunoglobulin receptor bound particles? The focus of this review is to provide an overview of the expression and function of various immunoglobulin receptors.

## 1. Chronic Inflammatory Lung Diseases

The most prominent chronic inflammatory diseases of the lung are asthma and chronic obstructive pulmonary disease (COPD). These two common diseases are a major burden for public health and affect over 500 million people worldwide (World Health Organization: WHO/NMH/CHP/CPM/05.4.). Other chronic inflammatory lung diseases are hypersensitivity pneumonitis which is caused by antigen exposure and lung fibrosis, the cause of which is unknown and therefore the pathology is widely uncertain [1–7]. To an unknown reason the prevalence of all chronic inflammatory lung diseases is on the rise especially in Asia [8, 9].

In Europe and the USA cigarette-smoke-induced COPD was death cause no. 4 in 2008 and with a further increasing prevalence it is expected to become death cause no. 3 within the next decade. COPD is characterized by chronic inflammation of the small airways, with similar pathologies known for asthma [10–12]. These include airway constriction, hyperplasia, and hypertrophy of mesenchymal cells, increased mucus production, tissue remodeling, and finally tissue degradation, emphysema [10, 11]. The latter pathology is regarded as a different disease by some investigators [10]. 

Asthma is the most frequent chronic lung inflammation in children and is the major cause of absence from school and work. Several studies indicated that the prevalence of asthma is increasing, especially in countries with an increasing urban life style [9, 13–15]. However, beside extensive investigations worldwide the link of urban lifestyle and asthma is not understood. Some studies suggest that countryside living and contact with animals of the mother during pregnancy and in early childhood may be protective [16, 17]. Therefore these studies indicate a central role of the innate immunity as well as of the adaptive immunity [18, 19]. 

However, the molecular or cell biological events that lead to the similar pathogenesis of chronic inflammatory lung diseases in distinct segments of the lung are not fully understood. Worse, there are no curative drugs available and only the symptoms can be controlled. Asthma symptoms can be reduced by inhaled glucocorticoids, long-acting *β*2-agonists, muscarinic receptor antagonists, or by leukotriene inhibitors, phosphodiesterase inhibitors, or anti-IgE antibodies [20–24]. Even there are an increasing number of drug classes being approved for asthma therapy from which all reduce the inflammation, but do not show any effect on the extensive airway remodeling which is well documented in childhood asthma [25–27]. 

In COPD there are not many therapeutic options besides inhaled glucocorticoid and long-acting *β*2-agonists, and their efficacy is low in most patients [12, 28]. The fact that remodeling of the airways persists after the environmental stimulus is gone, while inflammation depends on the presence of a stimulus, points out that the immune response may not always be the initiating factor for chronic inflammatory lung diseases [29, 30].

The current hypothesis is that chronic inflammatory lung diseases are causatively linked to an overreactive or out of control immune response to environmental factors [30, 31]. Asthma and other chronic inflammatory lung diseases can be induced by nonallergic factors such as cold air, humidity, exercise, or psychological stress [32–35]. Of course the immune system plays a major role in the pathogenesis of chronic inflammatory lung diseases, but there is evidence that it may not be the initiating factor. 

New investigations in primates and humans suggest two major events that predispose an individual to develop chronic inflammatory lung diseases during life: (i) maternal exposure to environmental factors that reorganize the lungs maturation during pregnancy and (ii) exposure to such factors in the first six years of life [29–31, 36–40]. The mechanism how maternal behavior or exposure to risk factors modifies the embryonic lung development may involve innate immunity or immunoglobulin synthesis [41, 42]. Interestingly one of the earliest signs of the lung's increased susceptibility to develop a chronic inflammatory disease is the remodeling of the large or small airways [25, 36, 37]. Unfortunately, we do not understand the mechanisms how environmental factors increase the susceptibility of the lung to develop chronic inflammation upon a second, independent (unknown) triggering event. Neither do we understand the disease specific mechanism(s) that leads to the wide range of clinical phenotypes, nor for the different age of onset, nor the gender specificity [43–45].  In the past two decades we accumulated knowledge of the immune response in chronic inflammatory lung diseases, but this knowledge did not help us to explain the entire pathology of asthma or COPD or of any other chronic inflammatory lung disease. 

## 2. The Immune Response of Tissue-Forming Cells

Tissue-forming cells have been excluded from studies of the immune response for a long time as it was assumed that these cell types do only respond to immunoglobulin indirectly through cytokines and growth factors which are released by activated immune cells after antigen or immunoglobulin binding. However, there is evidence that this is not the full story. Evidence for an active participation of tissue-forming cell in the lungs response to allergens is provided by a handful of research groups and thus the literature is rare. In allergic asthma and some forms of COPD the immune response is a central mechanism that initiates the pathology; however, we do not fully understand how it works. The immune response is a major cause of exacerbation in allergic asthma, but it cannot explain how nonallergic asthma is caused [32, 44, 46]. Asthma exacerbation can be induced by exercise, stress, humidity, cold or hot air [19, 46]. However, the immune response of the lung seems to be more complex than anticipated for the past decades. Besides immune cells the tissue-forming cells of the lung express immunoglobulin receptors and respond to immunoglobulins. 

**Figure 1:**
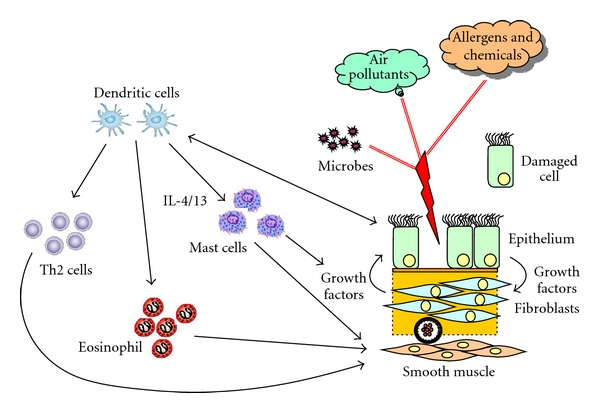
The network of interactions between tissue-forming resident airway cells and immune cells.

To understand the cause of chronic inflammatory lung diseases one has to include the contribution or the causative role of tissue-forming resident cell types which give the lung its structure and guarantee its function. In 1922 asthma was first described as a disease caused by overreactive airway smooth muscle cells and an increased size and number of smooth muscle bundles surrounding the airways [47]. Then the role of the immune cells and their response to environmental factors were regarded as being more important; however, recent studies refocused on the role of the smooth muscle and its interaction with other cell types [36, 48–52]. In COPD the disrupted interaction of epithelial cells with the submucosal fibroblasts has moved into the centre of attention in the past years [36, 53–56]. However, to identify the pathologic mechanism(s) that leads to chronic inflammatory lung diseases one has to understand the concerted interaction of all cell types with each other and this includes tissue-forming cells and immune cells (Figure 1).

 One important aspect of allergic chronic inflammation is the response of the lung to inhaled allergens. Therefore it is surprising that the fact that tissue-forming cells include bronchial epithelial cells, fibroblasts, and airway smooth muscle cells has drawn not much attention, while the response of immune cells was such much in the focus during the past decades. First reports on immunoglobulin receptor expression and function in tissue-forming resident lung cells were published in the 1980s, but did not trigger extended investigations. In the following I will summarize the knowledge of immunoglobulin receptor expression on tissue-forming cells of the human lung and their possible contribution to the pathogenesis of mainly asthma and COPD.

## 3. Epithelial Cells

Epithelial cells of the airway form the barrier between the tissue and the inhaled air. They are the first cell type which is exposed to inhaled allergens, dust particles, vapor or chemicals. The epithelial cell layer consists of two subtypes, the outer ones are ciliated cells which are considered as end-differentiated cells and which are shed off when they do not function properly [57, 58]. The outer ciliated epithelial cells are followed by a layer of basal epithelial cells, which are a type of precursor and can transform into goblet cells or ciliated epithelial cells [53, 58–60]. The basal epithelial cell layer is followed by the basement membrane which consists of pure extracellular matrix (ECM). It was assumed that the basal membrane forms an uninterrupted barrier between epithelial cells and subepithelial fibroblasts [45, 57]. However this view is challenged by several reports that indicate direct cell-cell contacts between epithelial cells and subepithelial fibroblasts [61–64]. If such direct cell-cell interactions between epithelial cells and submucosal fibroblasts exist, they must bridge the basal membrane and this may act as a direct passage of inhaled substances into the airway wall [65–68]. 

In asthma the number of columnar epithelial cells is shed off more frequently and the number of goblet cells is increased [59, 60]. Shedding may explain the reported increased epithelial cell fragility; however, to increase shedding additional factors must weaken the intercellular attachment of neighboring epithelial cells and the basal epithelial cells [57, 58, 61]. In COPD the direct epithelial cell-fibroblast interaction seems to be disrupted [36, 53–56]. It is also indicated that an abnormal expression or activity of adhesion molecules coupled with an alteration in the composition of the extracellular matrix (ECM) in asthma and COPD changes the response of the tissue-forming cells to antigens and Ig receptors [65, 67, 68]. 

Epithelial cells respond to inhaled particles and allergens not only through immune cell released cytokines, but also through cell membrane receptors [69–72]. Human bronchial epithelial cells express the low-affinity IgE receptor (CD23) as well as the high-affinity receptor and responded to its activation [73, 74]. The activation of the low-affinity IgE receptor resulted in secretion of endothelin-1, a well-known stimulator for fibrotic processes [74], and the activation of the high-affinity IgE receptor led to the release of 15-hydroxyeicosatetraenoic acid from epithelial cells of asthma patients only [73]. However, since this report by Campbell et al. no other study investigated the role and function of the low-affinity IgE receptor on epithelial cells. In epithelial cells of the intestine of allergic patients it had been shown that an allergen-IgE complex activated IL-8 secretion via the intracellular signal proteins Erk1/2 mitogen-activated protein kinase (MAPK) [75]. 

In an animal model it was suggested that allergen inhalation upregulates the expression of the polymeric Ig receptor by bronchial epithelial cells and its stimulation increased IgM and IgA secretion [76]. The study showed that this effect was paralleled by Th17 cell activation, but did not provide direct evidence for such a link. If such a mechanism could be confirmed in humans it would add significant weight to the regulation of immune response by tissue-forming cells. 

*In vitro* experiments in a rat model suggest that alveolar epithelial cells also are able to express the IgG receptor and its expression is affected by glucocorticoids [77]. In other species and epithelial cells of other organs than the lung it had also been reported that the IgG receptors are expressed and are functional [78, 79]. Importantly it was also shown that the predisposition to allergies can be mediated by breastfeeding through maternal IgG and its receptor expression on embryonic lung epithelial cells [80]. One study performed in rat epithelial cell monolayers suggested that IgG via the Fc receptor enables antigen to be transferred through the epithelium or the epithelial cell, respectively, unchanged and be secreted on the apical side to subepithelial mesenchymal cells [77]. This would be an important new mechanism which will change the thinking of allergen presentation and immune response in the lung if it could be proven in an animal model or in humans. 

**Figure 2:**
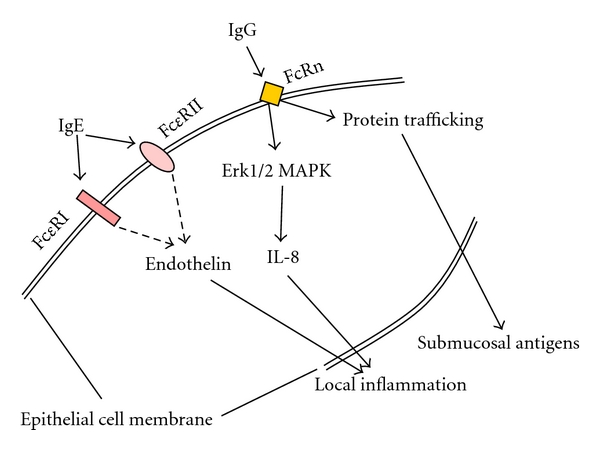
Epithelial cells express IgE and IgG receptors and respond directly to the respective immunoglobulins. Suggested (dashed line) and proven intracellular signaling pathways in human and animal airway epithelial cells. Importantly the IgG receptor expressed on airway epithelial cells may enable antigens to path un-changed through the epithelium and then contact with subepithelial mesenchymal cells [77].

Together these observations may be helpful to understand particle trafficking through the epithelium barrier in the lung and the contact of submucosal mesenchymal cells to such environmental factors [77, 81, 82]. The suggested functions of immunoglobulin receptors on epithelial cells are summarized in Figure 2. Unfortunately no studies have confirmed these data in humans. 

## 4. Airway Fibroblasts and Fibrocytes

Bronchial submucosal fibroblasts have been also reported to be involved in the response to environmental factors and to viral infection. The innate immune system was activated by rhinovirus infection in humans and induced interferon-g synthesis. Furthermore, it was shown that the virus reproduced in submucosal human bronchial fibroblasts [83]. In an ovalbumin inducible airway inflammation mouse model it was reported that submucosal fibrogenesis was induced by the allergen through a Smad3-dependent pathway; however the precise mechanism how the allergen activated the fibroblast type cells was not characterized [84]. Another study indicated that the increased submucosal airway remodeling upon allergen stimulation involves the recomposition of the ECM and is directed by the communication of epithelial cells and submucosal fibroblasts [85]. 

While no study reported the expression and function of any immunoglobulin receptor on lung fibroblasts there is indirect evidence that this cell type must express such receptors. Knight et al. reported in 1999 that IgE as well as IL-1*β* stimulation increased leukemia inhibitory factor (LIF) and its receptor (LIFR) in human lung tissue, with the highest expression in fibroblasts [86]. However, since these experiments were performed in isolated tissue sections it might be that the response of the fibroblasts involved their activation by mast cells. 

Fibrocytes that were differentiated from CD14^+^ blood cells and exposure to serum amyloid P, which bound to the IgG receptor inhibited the signalling leading to fibrocyte differentiation. Furthermore, monoclonal antibodies binding to IgG receptor I (CD64) or II (CD32) also inhibit fibrocyte differentiation, indicating that tissue structure can be directly modified by immunoglobulins [87]. 

## 5. Airway Smooth Muscle Cells

Bronchial or airway smooth muscle cells belong to the best studied tissue-forming resident cells in asthma and COPD. As mentioned above asthma was first be considered as a disease of the airway smooth muscle [47]. Most if not all asthma patients show a significant increase of airway smooth muscle bundles and cell numbers within the bundles [25, 36, 50]. Investigations in childhood asthma and a more recent asthma model in rhesus monkeys suggest that tissue remodeling occurs already during pregnancy and is further increased during the first 6 years of life: mostly before any sign of inflammation can be found [25, 36, 50, 88–92]. Other studies even suggest that the exposure to environmental factors during pregnancy starts a mechanism that deregulates the lung maturation and predisposes the embryo to develop chronic inflammatory lung diseases later in life [26, 36–41, 92, 93]. 

In regard to COPD the exposure to cigarette smoke seems to be an essential trigger; however, what sets the lung to develop a chronic inflammation as a response to allergic asthma stimuli is not known [39, 40, 44, 94, 95]. In asthma models in rhesus monkeys the significant structural change of the airway smooth muscle cell bundles around the airways of house dust mite and ozone-challenged animal is impressive [92]. This observation is that in asthma the smooth muscle cell bundles arrange in a spiral-like structure which constricts the airways much more forcefully than the randomly arranged muscle bundles in a healthy airway [92]. This leads back to some questions from the late Professor A. Woolcock (Sydney University Royal Prince Alfred Hospital, Sydney, Australia) to her students. What is the function of the smooth muscle bundles in the normal lung? Why do we need smooth muscle bundles around the airways? These questions have never been answered.

Among the new classes of asthma therapies are antibodies that bind to IgE and neutralize it [24]. Anti-IgE antibodies bind to the IgE receptor docking site and thus prevent binding of IgE to its receptors. Recent studies indicate that this new class of asthma drugs seems to be very effective in asthma therapy and significantly reduces symptoms and the need of other medications [96, 97]. It would be too easy to argue that the beneficial effect of the anti-IgE antibodies is achieved by the disruption of the IgE-IgE receptor interaction of immune cells. 

There are several studies starting from the late 1990s that provide evidence that the airway smooth muscle cell expresses and responds to the low as well as to the high IgE affinity receptors [98–103]. Furthermore, there is evidence that the expression of the IgE receptors is increased in asthma [102, 103]. Inhibition of IgE significantly reduced the secretion of proinflammatory cytokines by airway smooth muscle cells, and it also reduced their proliferation, at least *in vitro *[104–106]. Furthermore, it has recently been shown that IgE activates the transcription and expression of the thymic stromal lymphopoietin in human airway smooth muscle cells, a factor which is important to the recruitment of circulating inflammatory cells into the lung [107]. This finding supports the idea that the tissue-forming cells of the airway wall significantly contribute to the immune response and that they may be the first cells in the line of defense, which are; however, deregulated in chronic inflammatory lung diseases. 

In response to IgE stimulation airway smooth muscle cells have been shown to secrete proinflammatory cytokines including IL-8, and eotaxin, which will attract neutrophils and granulocytes to infiltrate the airway wall and thereby extend the local inflammation [99, 101, 108]. These observations are in line with clinical studies showing an anti-inflammatory effect of anti-IgE antibodies. In contrast Xia et al. [109] did not find the expression of any IgE receptor on tissue-forming cells in tissue sections. This discrepancy to other studies [98–103] may be explained by different methods or antibodies to detect IgE receptors. Further studies have to reassess the tissue compartmental expression of Ig receptors in the healthy and diseased lung.

**Figure 3:**
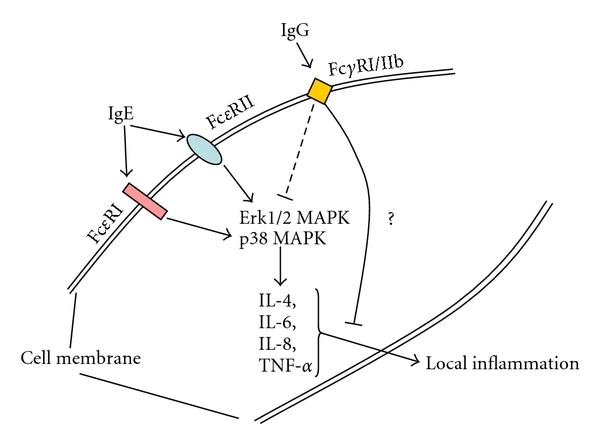
Expression and function of immunoglobulins and their receptors on human airway smooth muscle cells. Likely interaction and crosstalk of Ig receptors through shared intracellular signaling pathways. Reported pathways by which Ig receptors modulate cytokine synthesis by tissue-forming cells in chronic inflammatory lung diseases.

Recently it was reported that human airway smooth muscle cells also express the IgG receptors, Fc*γ*Rs-I, and -IIb [109]. Furthermore, the activation by IgG downregulated IL-1*α* induced cytokine production and reduced the activation of the two signaling proteins Erk1/2 MAPK and p38 MAPK. In sharp contrast to other studies the authors did not find any expression of the IgE receptors in their isolated cells. No reports are available for the expression of other immunoglobulin receptors on human airway smooth muscle cells. The possible function of the immunoglobulin receptors on human airway smooth muscle cells is summarized in Figure 3.

The above scarce data is evidence that the role of tissue-forming resident cell activation by immunoglobulins, not only IgE, must be investigated in more detail.

## 6. Conclusion

Tissue-forming cells of the human lung express and respond to the activation of at least two immunoglobulin receptors, IgG and IgE receptors. Thereby they directly contribute directly to inhale environmental factor such as allergens or dust without the need to activate immune reactive cells. The mechanism(s) that induce and control the expression of immunoglobulin receptor in these cell types have not been studied extensively. The relevance of the *in vitro* data on immunoglobulin receptor expression by tissue-forming resident lung cells has to be confirmed *in vivo* and its relevance to the pathologies of chronic inflammatory lung diseases has to be demonstrated. Ignoring the fact that these cells are an active participant in the lung's immune response to inhaled environmental factors will only delay the search for a better understanding of the distinct pathologies of the different chronic inflammatory lung diseases, and it will delay the search for new therapeutic strategies.
